# Exosomes biogenesis was increased in metformin-treated human ovary cancer cells; possibly to mediate resistance

**DOI:** 10.1186/s12935-024-03312-6

**Published:** 2024-04-16

**Authors:** Reza Abbasi, Vahid Nejati, Jafar Rezaie

**Affiliations:** 1 Department of Biology, Urmia University, Urmia, Iran; 2 Solid Tumor Research Center, Cellular and Molecular Medicine Research Institute, Urmia University of Medical Sciences, Urmia, Iran

**Keywords:** Exosomes, A2780 cells, Metformin, Ovary cancer, Skov-3 cells

## Abstract

**Background:**

Exosomes derived from tumor cells contribute to the pathogenesis of cancers. Metformin, the most usually used drug for type 2 diabetes, has been frequently investigated for anticancer effects. Here, we examined whether metformin affects exosomes signaling in human ovary cancer cells in vitro.

**Methods:**

Human ovary cancer cells, including A2780 and Skov3 cells, were treated with metformin for either 24–48 h. Cell viability and caspase-3 activity were determined by MTT (3-[4,5-dimethylthiazol-2-yl]-2,5 diphenyl tetrazolium bromide) and colorimetric assays respectively. Oil-Red-O staining and in vitro, scratch assays were used to examine cellular toxicity and wound healing rate. After treatment with metformin, exosomes were isolated from cells and quantified by acetylcholinesterase (AChE) assay, Dynamic Light Scattering (DLS), and their markers. Genes related to exosomes signaling were analyzed by real-time PCR or western blotting.

**Results:**

Our results showed that metformin decreased the viability of both cells dose/time-dependently (*P* < 0.05). Metformin increased the activity of caspase-3 (*P* < 0.05) as well as the number of Oil-Red-O positive cells in both cell lines. In vitro scratch assay showed that the cell migration rate of metformin-treated cells was decreased (*P* < 0.05), whereas AChE activity of exosomes from metformin-treated cells was increased (*P* < 0.05). Concurrent with an increase in CD63 protein levels, expression of Alix, CD63, CD81, Lamp-2, and Rab27b up-regulated in treated cells (*P* < 0.05).

**Conclusion:**

Results indicated that metformin had a cytotoxic effect on ovary cancer cells and enhanced exosome biogenesis and secretion.

## Introduction

Extracellular vesicles (EVs) such as exosomes refer to cell-derived vesicles that contain various types of proteins, RNA, lipids, DNA, metabolites, and carbohydrates [1]. Exosomes biology, a rapidly growing field of research, has gained scientists’ attention over the past few decades due to its pivotal role in regulating cellular processes [1]. They transfer biomolecules to target cells and, therefore participate in pathological conditions like cancer [2]. A growing body of evidence shows exosomes are pivotal factors for tumorigenesis, invasion, metastasis, drug resistance, and even tumor angiogenesis [3, 4]. Exosomes can be used as biomarkers for different diseases such as cancers. As exosomes are present in most body fluids, they can be isolated non-invasively, therefore, their content such as RNAs and proteins can be used for prognostic and diagnostic purposes [5]. Exosomes derived from tumor contain exact information about tumor status [5]. In addition, cell status may affect the level of exosomes production and content. For example, various drugs and agents can affect exosomes/EVs signaling in cells [6]. Confirmed that exosomes from tumor cells actively medicate metastasis through the formation of primary pre-niche in the site of metastasis [7]. Exosomes produced by ovarian cancer cells have been shown to promote tumorigenesis [8]. For example, exosomes from ovarian cancer can inhibit T-cell, which causes immune escape [9]. It was demonstrated that exosomes from ovarian cancer cells contain cleavage of L1 that can promote cell migration and regulate tumor cell function [10]. Clancy et al. reported that exosomes collected from malignant ascites of patients with ovarian cancer can transfer membrane-type 1 matrix metalloproteinase (MMP) to the cells that stimulate ovarian cancer invasion [11]. Ovarian cancer stands as one of the most prevalent malignancies within the female reproductive system and ranks as the fifth leading cause of cancer-related fatalities in women globally [12]. A growing body of experiments has shown inhibition of exosomes/EVs biogenesis and secretion may be a possible way to inhibit tumorigenesis [13]. Metformin (1,1-dimethylbiguanide hydrochloride), used in patients with type 2 diabetes, influences the tumor microenvironment, disrupting communication between the tumor and its surroundings [14]. Several cohort studies have revealed a notable connection between metformin usage and improved survival among cancer patients. Recent research has demonstrated this effect on endothelial cells and ovarian cancer-associated fibroblasts (CAFs) [15–17]. Metformin was found to inhibit the migration of ovarian cancer cells through the AMP-activated protein kinase (AMPK) pathway, which reduces a specific histone modification Histone H3 at lysine 27 (H3K27me3) [18]. It was demonstrated that metformin exerts anticancer effects on ovarian cancer cells by inhibiting MSLN-mediated interlukin6 (IL6)/ Signal Transducer and Activator Protein (STAT3) signaling, along with downregulation of vascular endothelial growth factor (VEGF) and transforming Growth Factor beta1 (TGFb1) [19]. Despite exosomes significance in ovary cancer, there is no information related to the modulation of exosomes signaling in ovary cancer cells following treatment with metformin. In this study, we additionally assessed the hypothesis, of whether metformin could alter exosomes biogenesis and secretion in two ovary cancer cells.

## Materials and methods

### Cell culture

The human ovarian cancer cell lines (A2780 and Skov3 cells) were purchased from the Iranian National Cell Bank (Pasture Institute, Iran) and cultured in RPMI 1640 (Biosera) enriched with 10% fetal bovine serum (FBS, Gibco) and 1% streptomycin/ penicillin. The cell culture plates and flasks were kept in an incubator set at 95% humidity, 37 C, and 5% CO2. Conditional medium was replaced each 2–3 days with fresh medium. At 80% confluency, cells were passaged using 0.25% trypsin-EDTA (Gibco).

### Treatment protocol

Metformin (Sigma-Aldrich) was dissolved in a cell culture medium for downstream treatment. For cell viability assessment, cells were treated with serial concentrations of metformin (5 mM, 10 mM, 20 mM, and 40 mM). For downstream experiments, cells were incubated with the IC50 (Inhibitory Concentration at 50%) value of metformin and kept as Met group. One group was kept as a control group with the same condition that did not receive metformin.

### MTT assay

Cell viability was assessed using the MTT colorimetric assay, which measures cellular metabolic activity. In brief, A2780 and Skov3 cells (7 × 10^3^ cells per well) were initially cultured into a 96-well plate for 24 h. Next, the culture medium was removed, and different concentrations of metformin (5 mM, 10 mM, 20 mM, and 40 mM) were added into the relevant wells for 24 h and 48 h. Following treatment, MTT reagent (5 mg/ml; Sigma-Aldrich) was added and formazan crystals were dissolved using 100 µl DMSO (Sigma-Aldrich). Absorbance density (OD) was recorded using a microplate reader (TECAN/SWISS) at 570 nm. Cell viability was reported as the percentage of OD treatment cells against OD control cells. IC50 was calculated using OD values and Graph Pad 8 (Prism) software.

### Caspase3/CPP32 colorimetric assay

Caspase-3 enzyme activity was examined using a commercial kit (Can.Lot60206, BioVision) according to the recommendation. In brief, 1 × 10^6^ cells were treated with metformin for 48 h and then lysed with lysis buffer to obtain supernatant as proteins. For each group, 50 µg of protein was mixed with 50 µl of 2X Reaction buffer (containing 10 mm DDT) and 5 µl DEVD-pNA. The mixtures were kept for 90 min at 37 °C. Finally, absorbance was measured at 405 nm using a microplate reader (TECAN/SWISS).

### Oil-Red- O staining

Briefly, after the treatment protocol, 5 × 10^4^ cells were washed with PBS three times and incubated with methanol for 15 min at room temperature, and then fixed in paraformaldehyde solution (PFA, 4%) for 20 min. After washing, cells were kept in Oil Red O staining solution (Sigma-Aldrich) (0.1%) for 30 min. Finally, after washing, images were captured by light microscopy (IM-3/ OPTIKA Italy) equipped with a CCD camera (TrueChrome II).

### In vitro scratch assay

In vitro, scratch assay was used to monitor the movement of A2780 and Skvo3 cells after treatment with metformin. Cells (4 × 10^5^) were seeded onto a six-well plate and grown until they formed a complete monolayer. Then, a straight-line wound was created by gently scraping the surface of the culture dish. Images of the wound area were captured three times (0 h, 24 h, and 48 h) using light microscopy (IM-3/ OPTIKA) equipped with a CCD camera (TrueChrome II). The percentage cell migration rate was measured using Image J software (ver. 1.44p) and calculated as healing rate (%) = (New scratch area - second scratch area)/ New scratch area × 100.

### Exosome isolation

To isolate exosomes from the cell culture supernatant of both A2780 and Skov3 cells, we used a commercial exosomes isolation kit (Cat no: #3603 − 100; Cibbiotech). After treatment with metformin, cells continued 48 h starvation, supernatants were collected and centrifuged at 1500 g for 5 min to remove cell debris. Next, according to the kit’s recommendation, reagent A was mixed with supernatants (1:5 ratio) overnight at 4 °C. In the final step, samples were centrifuged at 14,000 g for 40 min at the 4 °C. Then, exosome pellets were appeared and resuspended in 100 µl of reagent B and stored or used.

### Quantification of exosome

For this purpose, we used a commercial cholinesterase kit (Cat no. BXC080, Biorexfars) to investigate AChE activity according to the manufacturer’s recommendation. In brief, reagent 1 was added to exosome samples and kept for 5 min at 24 °C. After mixing with reagent 2, absorbance values were recorded at 405 nm at three intervals using a microplate reader system (TECAN/SWISS). AChE activity was measured using a formula: AChE activity (U/l) = ΔAbs/min × 65,800.

### Dynamic light scattering (DLS)

To investigate the size distribution of isolated exosomes, a 100 µl exosomes sample was diluted with 1 mL PBS before injecting it into the system (Nano ZS ZEN 3600, Malvern Panalytical Ltd, Malvern, UK). Exosomes size was measured by a laser system at a wavelength of 633 nm at 25 °C. Data analysis was carried out using Zetasizer software version 6.0.

### Western blotting

To measure exosome markers on isolated exosomes, we used western blotting analysis to detect CD63 protein on exosomes. Western blotting was completed as previously described [20] using primary antibody CD63 (MX-49.129.5; sc-5275; Santa Cruz Co) and secondary antibody (m-IgGκ BP-HRP; sc-516,102; Santa Cruz Co ).

### RNA extraction

To extract total RNA from the experimental groups (control cells and metformin-treated cells), we used an RNA extraction kit (cat.no: FARBK001; Favorgen). Briefly, 350 ml FARP and 3.5 ml B-Mercaptoethanol were added into cells and kept for 5 min. Then, the samples were placed into a filter column and centrifuged at 18,000 g for 2 min at 4 °C. The resulting solution was mixed with 1 volume of 70% ethanol and transferred into a new filter column. After centrifuging at 18,000 g for 2 min at 4 °C, wash buffer 1 and wash buffer 2 were added into the filter column and centrifuged again for 1 min at 4 °C. To remove any remaining liquid, filter columns were centrifuged for 3 min. Finally, 100 ml ddH2O was added and centrifuged for 1 min at 4 °C. The extracted RNAs were analyzed for their purity and concentration using a nanodrop system (EPOCH/BioTek) for downstream experiments.

### cDNA and real time PCR

We used real-time PCR analysis to evaluate the expression of exosomal gens. For this purpose, we initially constructed cDNA strands according to the First Strand cDNA Synthesis kit’s recommendation (Cat no: YT4500, Yekta Tajhiz Azma Co). Next, cDNA samples were used to investigate the expression levels of genes using SYBR Green PCR Master Mix (Cat no: YT2551, Yekta Tajhiz Azma Co) and a MIC Real-Time PCR System (Swiss). The real-time PCR program was: 95 °C for 5 s, 95 °C for 10 s, 59/63°C for 30 s, and 72 °C for 20 s set on 45 cycles. GAPDH was kept as the internal control. The relative expression levels of genes were measured by the comparative 2 (−ΔΔCT) method. The primers used in this study are listed in Table 1.

**Table 1:** List of primers

Gens	Forward	Reverse	Tm
Lamp-2	GGCAATGATACTTGTCTGCTGGC	GTAGAGCAGTGTGAGAACGGCA	63
Rab27a	AGAGGAGGAAGCCATAGCAC	CATGACCATTTGATCGCACCAC	59
Rab27b	GGAACTGGCTGACAAATATGG	CAGTATCAGGGATTTGTGTCTT	59
Alix	CTGGAAGGATGCTTTCGATAAAGG	AGGCTGCACAATTGAACAACAC	63
CD63	TCCTGAGTCAGACCATAATCC	GATGGCAAACGTGATCATAAG	63
CD81	CTGCTTTGACCACCTCAGTGCT	TGGCAGCAATGCCGATGAGGTA	63
GAPDH	ACATCGCTCAGACACCATG	TGTAGTTGAGGTCAATGAAGGG	59

### Western blotting for cellular CD63

After the treatment protocol, cells were gently mixed with RIPA buffer (Sigma) while keeping everything cold. To collect protein, the samples were centrifuged at 15,000 g for 20 min at 4 °C. A nanodrop system (Biotek) was used to determine amounts of protein. Then, equal amounts of the total protein (100 mg) were separated by SDS-polyacrylamide gel electrophoresis (SDS-PAGE10%). Next, proteins were transferred into PVDF membrane (0.2 millimeters, Millipore). Membranes incubated with 5% bovine serum albumin (BSA) in TBST for 1 h. After washing with TBST, primary anti-human CD63 antibodies (MX-49.129.5; sc-5275; Santa Cruz Co) were incubated with the membrane overnight at 4 °C. After washing with TBST three times, anti-mouse HRP (m-IgGκ BP-HRP; sc-516,102; Santa Cruz Co) were added for 1 h at room temperature. In the final step, to visualize the protein bands, the ECL reagent (Roche) was added and the intensity of the bands was measured using NIH Image J software (ver. 1.47p).

### Statistical analysis

Results were mean ± S.D. and analyzed using either one-way ANOVA with Tukey’s post-hoc test or t-test. Data analysis was completed using GraphPad Prism version 9.0 (GraphPad Software, Inc.). For all tests, *P* < 0.05 was considered statistically significant between different groups. In figures, * means *P* < 0.05.

## Results

### Cell viability

**Fig. 1:**
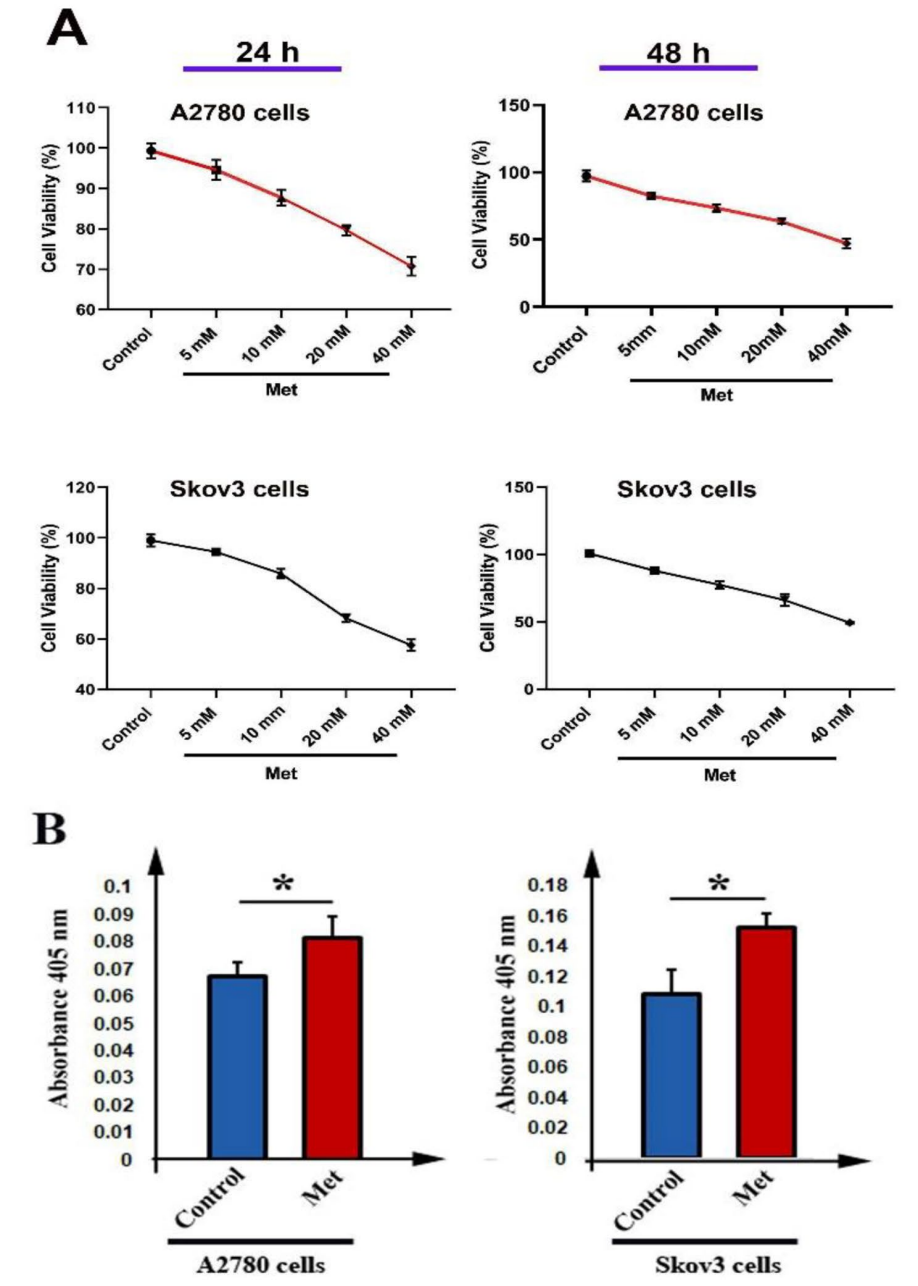
MTT assay for A2780 and Skov3 cells after 24 h and 48 h treatment with different concentrations of metformin (**A**). Caspase-3 activity of A2780 and Skov3 cells treated with metformin after 48 h (**B**). ANOVA and Tukey’s test for MTT assay and t-test for caspase-3 activity. *n* = 3, * *P* < 0.05

To investigate the cytotoxic effect of metformin on human ovarian cancer cells, we performed a simple MTT assay. Results showed that metformin decreased the viability of A2780 and Skov3 cells after either 24–48 h treatment (Fig. 1A). As shown in Fig. 1A, a decrease in the cell viability depended on dose and time of incubation. Furthermore, we found that the IC50 value of metformin for A2780 and Skov3 cells was 12.05 mM and 16.14 mM for 24 h, and 10.09 mM and 10.89 mM for 48 h respectively (Table 2).

**Table 2:** IC50 values of metformin for A2780 and Skov3 cells

	IC50			
Cells	A2780 cells		Skov3 cells	
Time	24 h	48 h	24 h	48 h
Concentration	12.05 mM	10.09 mM	16.14 mM	10.89 mM
R squared	0.97	0.98	0.97	0.97
Degrees of Freedom	12	13	13	13

### The activity of caspase-3

To explore the apoptosis rate of cells, we used colorimetric assay to measure caspase-3 activity. Cells were treated with metformin for 48 h. We found that the activity of caspase-3 in both A2780 and Skov3 cells was significantly increased compared to control cells (*P* < 0.05, Fig. 1B).

### Oil red O staining

**Fig. 2:**
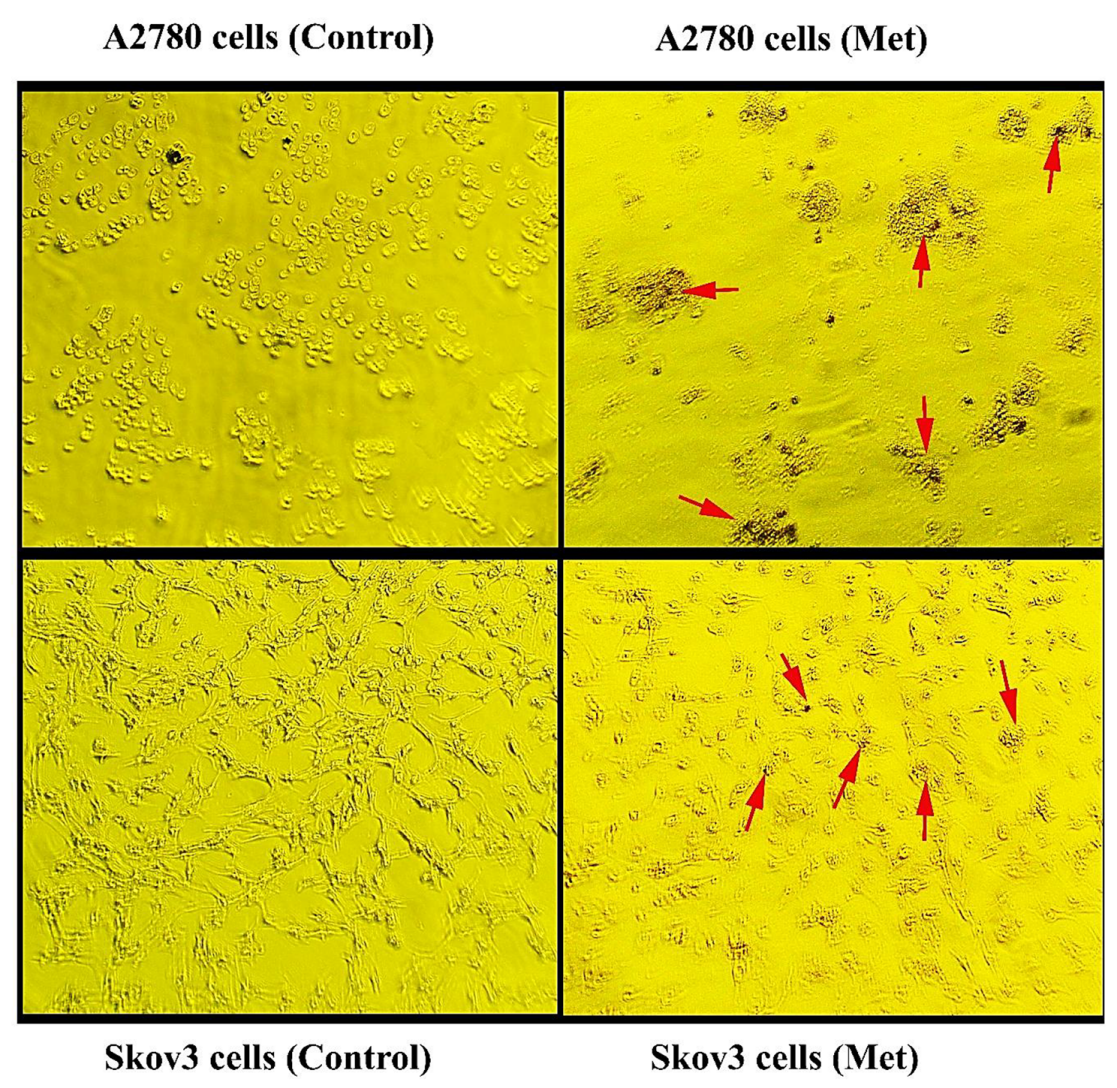
Oil Red-O staining for A2780 and Skov3 cells. Arrows show Oil Red-O positive cells in treated cells (Met group). Magnification ×10

To further scrutinise the cytotoxic effect of metformin, we accomplished the Oil Red O staining assay. As shown in Fig. 2, we found many Oil Red O positive cells in metformin-treated cells. This may suggest the accumulation of intracellular lipid drops in cells and lipotoxicity.

### The cell migration rate

**Fig. 3:**
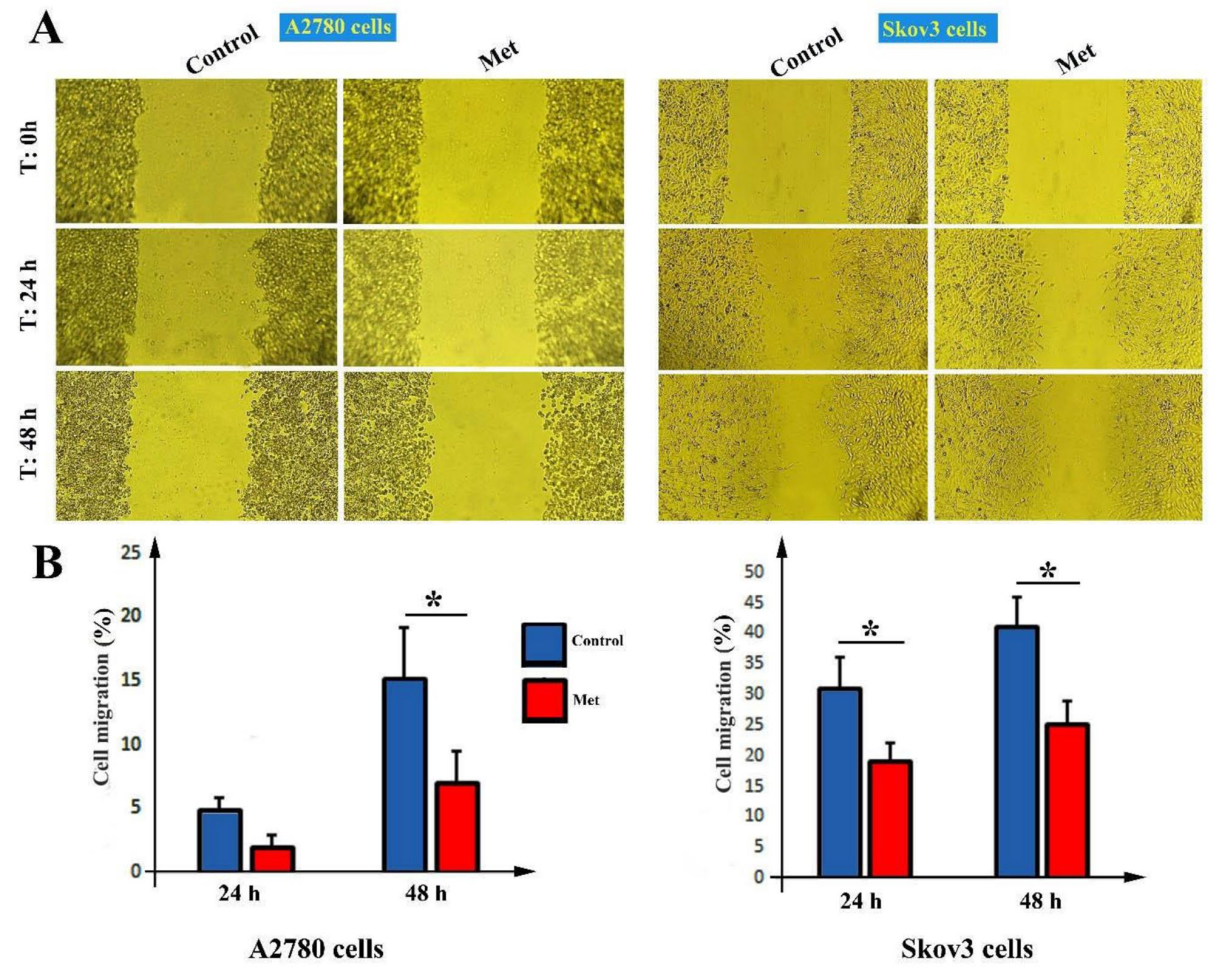
In vitro scratch assay for A2780 and Skov3 cells after 0 h, 24 h, and 48 h incubation with metformin (**A**). Percentage of cell migration rate in A2780 and Skov3 cells after 0 h, 24 h, and 48 h incubation with metformin (**B**). Magnification ×10

To explore the cell migration rate of A2780 and Skov3 cells, in vitro scratch assay was used (Fig. 3A). Result showed that the wound healing rate of treated A2780 cells and control was not altered after 24 h (*P* > 0.05) (Fig. 3B). The wound healing rate of treated A2780 cells (Met group) was significantly decreased (8.1 ± 1 *v.s* 2.2 ± 1%) compared to control cells after 48 h post-treatment (*P* < 0.05, Fig. 3B). Similarly, compared to control cells, the wound healing rate of treated Skov3 cells was significantly decreased after 24 h (31 ± 5 *v.s* 20 ± 3%) and 48 h (41 ± 5 *v.s* 25 ± 4%) incubation with metformin (*P* < 0.05, Fig. 3B). In contrast, there was no significant difference between control and metformin treated cells (Met) (*P* > 0.05).

### Exosomes characterization

**Fig. 4:**
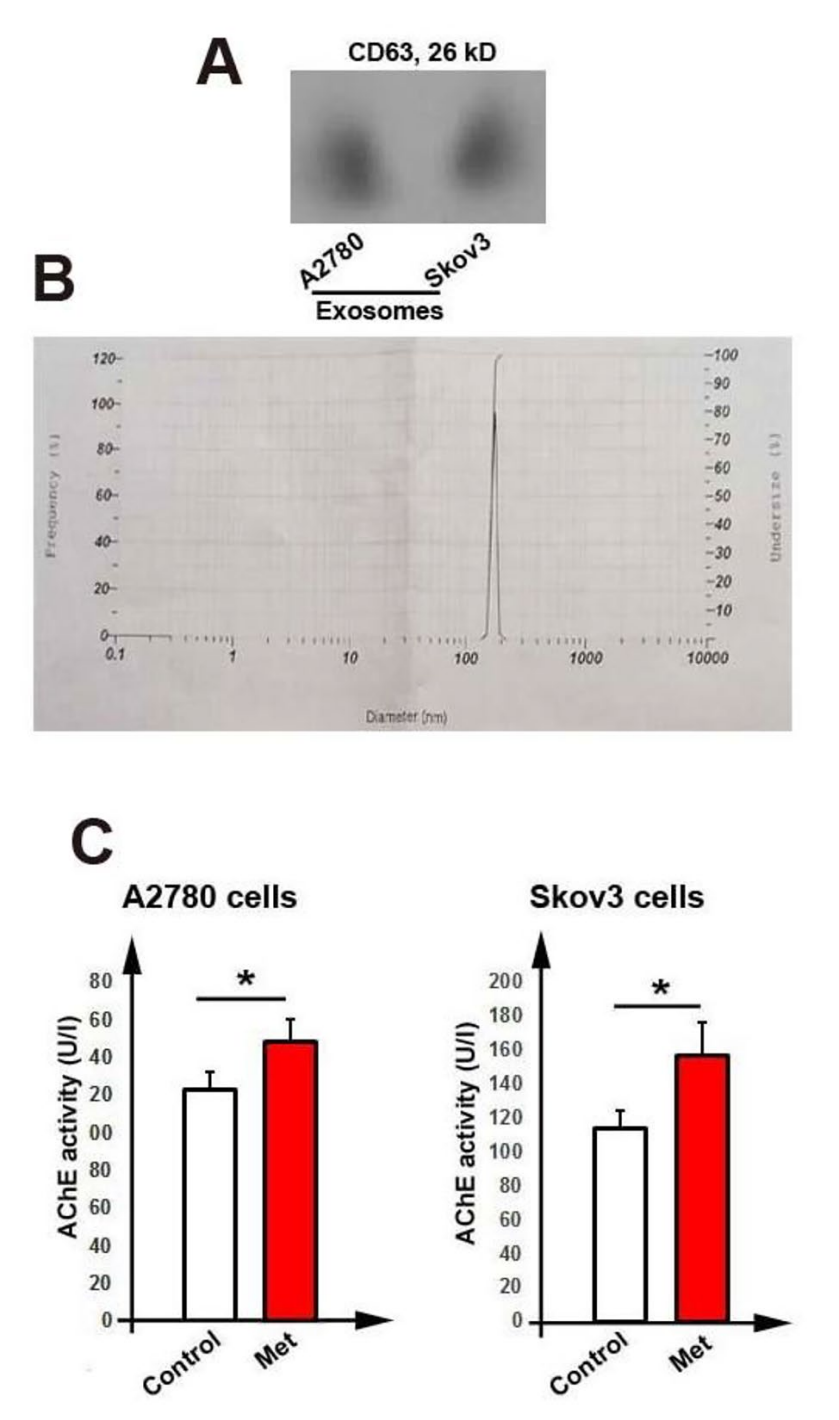
Western blotting analysis for the exosomal CD63 (**A**). The size distribution of isolated exosomes was analyzed by dynamic light scattering (DLS) (**B**). Acetylcholinesterase assay (AChE assay) for isolated exosomes (**C**)

For further examination, after the isolation of exosomes from cancer cells, we detected the exosomal marker CD63 by western blotting (Fig. 4A). We also performed a DLS assay for exosomes size distribution, which confirmed the average size of exosomes was between 110 and 150 nm (Fig. 4B).

### AChE activity of exosomes

AChE activity was used to measure the amount of exosomes released by cells. Results showed that AChE activity was increased in cells treated with metformin (Met) in both cell lines (*P* < 0.05, Fig. 4C). For example, AChE activity of A2780 was 149.11 ± 11 U/l against control (123.23 ± 9 U/l) and Skov3 cells was 157 ± 19 U/l against control (114 ± 11 U/l) (*P* < 0.05, Fig. 4C).

### The expression of exosomal genes

**Fig. 5:**
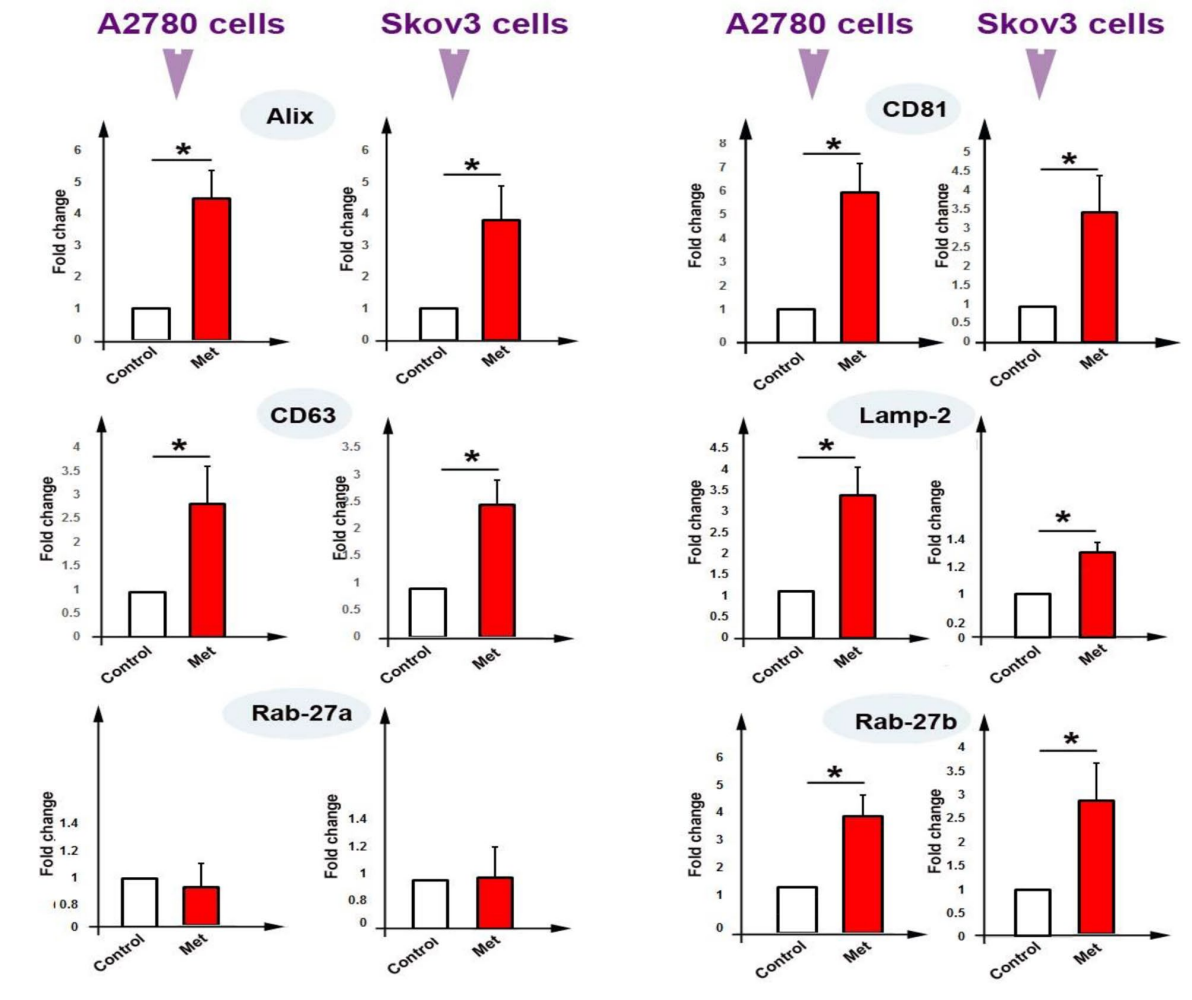
Real-time PCR assay for expression of genes including Alix, CD81, CD63, Lamp-2, Rab27a, and Rab27b in both A2780 and Skov3 cells. T-test. *n* = 3, * *P* < 0.05

Real-time PCR was used for measuring the expression of genes including Alix, CD81, CD63, Lamp-2, Rab27a, and Rab27b in both A2780 and Skov3 cells. At the mRNA level, as shown in Fig. 5, compared to control cells, the expression of Alix, CD81, CD63, Lamp-2, and Rab27b were up-regulated in both A2780 and Skov3 cells (*P* < 0.05). In addition, the expression of Rab27a was not significantly altered in treated cells compared to control cells (*P* > 0.05, Fig. 5). These results propose that metformin-induced exosome biogenesis and secretion by up-regulating key genes.

### The protein level of CD63

**Fig. 6:**
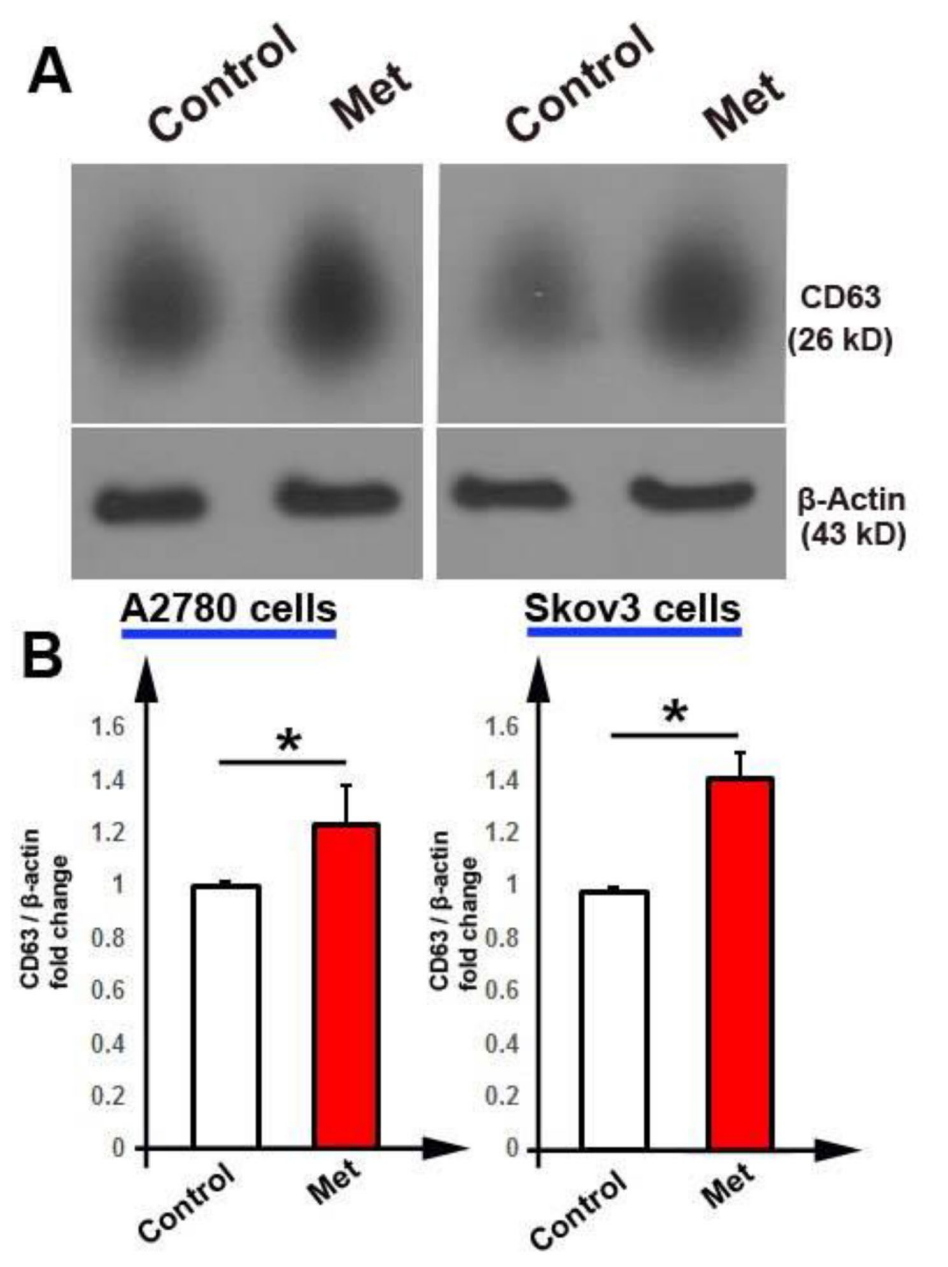
Western blotting analysis for CD63 protein expression in A2780 and Skov3 cells (A). Expression levels of CD63 protein were increased in Met groups (B). T-test. *n* = 3, * *P* < 0.05

To investigate the effect of metformin on the protein level of CD63 (an exosome marker), we completed western blotting using the SDS-PAGE process. Similar to the mRNA level of CD63 in cells, the raised expression level of CD63 at the protein level was also detected in treated cells. Our data show that the protein level of CD63 in Met groups increased as compared to the control group (*P* < 0.05; Fig. 6A and B).

## Discussion

Recent studies have shown that metformin has anticancer properties against different cancers [21]. Firstly, this study evaluated the cytotoxic effect of metformin on human ovary cancer cells. Furthermore, the association between metformin treatment and exosomes signaling has been examined in this study. The study of exosome dynamics is important in cancer therapy because exosomes are involved in cancer development and resistance. First, we found that metformin decreased the viability of both cells. Our findings were consistent with previous results, which indicated that metformin decreases the viability of cancer cells [22, 23]. In addition, we found that metformin increased the activity of caspase-3 in both cells, indicating an increase in apoptosis [24–26]. Caspase-3, a key component involved in the apoptosis process, plays a pivotal role in both the extrinsic and intrinsic apoptotic pathways [27, 28]. Although we did not assay to discover the exact mechanism, however, there is evidence that metformin could inhibit cell proliferation by activating AMPK [29], induce apoptosis, and decrease cell migration and invasion in a dose-dependent manner in ovary cancer cells [30, 31]. Another evidence for the cellular toxicity comes from our Oil-Red-O staining test which found that the number of cells positive to Oil-Red-O was increased in metformin-treated cells, suggesting lipid accumulation inside the cytoplasm and lipotoxicity [32]. In addition, we investigated the cell migration rate of cells treated with metformin; we found that metformin decreased the cell migration rate of both cells after 24 and 48 h post-treatment. This observation may correlate with a decrease in the migration ability of cells following exposure to metformin [33, 34]. Cancer metastasis is the hallmark of tumor development and we showed an inhibitory impact of metformin on cancer cell migration [34, 35].

We also performed an analysis for exosomes signaling if metformin affects their biogenesis and secretion in either A2780 cells or Skov3 cells. Our data showed a significant increase in AChE activity, indicating an elevated level of exosome secretion and biogenesis in cells treated with metformin [36]. These results are in agreement with the results of Soraya et al., indicating that metformin induces exosome secretion in U87MG cells [37]. We seek to know how metformin induces exosome biogenesis and secretion in cancer cells; and found that the mRNA level of genes related to exosome biogenesis and secretion such as Alix, Rab27b, CD81, CD63, and Lamp-2 concomitantly with CD63 protein level up-regulated in cells treated with metformin. CD63 protein contribute to exosomes biogenesis and loding. This protein is frequently used as exosomal marker [38]. A significant increase in the expression of these genes may correlate with an increase in exosomes biogenesis and secretion [39]. Our findings are consistent with previous results, for example, a study reported an up-regulation of Alix and CD63 in mesenchymal stem cells treated with metformin [40]. We found that the expression of Rab27a was not changed in treated cells. Reversely, it was previously reported that expression of this gene was increased in U87MG cells following incubation with metformin [37]. In addition, Feng et al. showed a downregulation rate in the expression of Rab27a in the MDA-MB-231 cells treated with metformin [41]. This discrepancy may relate to the type of cells. It was suggested that an increase in heparanase levels can trigger exosome secretion, and this process requires enzymatically active heparanase [42]. Vishnu et al. demonstrated that exposure of myeloma cells to cytotoxic drugs enhanced heparanase expression and release from cells [43].

Overall, previous studies have demonstrated that pre-conditioning of cells can alter exosomes secretion and content [44]. It seems that treatment with metformin causes an increase in exosomes biogenesis in ovarian cancer cells. Thus, an increase in AChE activity coincided with an up-regulation of exosomal genes and protein level of CD63, which may correlate with increased exosomes secretion from treated cells. This hypothesis confirms a previous study [37]. However, it is unclear why cells increase exosomes production in this condition. In our opinion, a possible mechanism is that cells compensate removal process by fusing multivesicular bodies (MVBs) with the plasma membrane [39]. MVBs are a subtype of endosomal compartments in which exosomes are formed [39]. There is evidence that exosomes/MVBs have crosstalk with other signaling pathways such as autophagy and lysosomal degradation pathways [45]. MVBs may participate in the removal of damaged biomolecules. Therefore, an increase in the dynamic of exosomes secretion may correlated with cellular damage and cytotoxic conditions caused by metformin as metformin can affect other signalings. Therefore, cells may be resistant to metformin cytotoxicity via increasing exosomes, and possibly inducing bystander effects [46].To confirm this, further studies are needed to investigate other signaling pathways concurrent with exosomes signaling. The present study has only investigated the biogenesis of exosomes in cells. Therefore, it is recommended that the content of exosomes should be investigated to answer questions like; Does metformin change exosomes cargo? What is the effect of exosomes from treated cells on other cells such as normal or cancer cells? Results so far have been very promising regarding molecular mechanism behind exosomes pathway in cancer cells treated with metformin. For clinical application, further studies on in vivo model are recommended because although metformin showed cytotoxic effects against cancer cells, at the same time, it increased exosomes biogenesis that may contribute to support tumor resistance and metastasis.

## Conclusions

From the outcome of our investigation, it is possible to conclude that exosomes biogenesis and secretion were increased following incubation with metformin. Regarding the cytotoxic effects of metformin, seemingly, exosomes signaling may crosstalk with another signaling to lessen cell damage. More research into the dynamics of exosomes is still necessary before obtaining a definitive answer to this hypothesis. In addition, we hope that our research will help uncover the effects of anti-cancer therapies on cellular signaling like exosomes. Exosomes may contribute to drug resistance. At the same time, we believe that further in vivo experiments are essential to confirm our results. For clinical application, additional experimental investigations are needed to uncover exosomes cargo and their effect on neighboring cells and cells located far from tumor location since exosomes travel throughout the body.

## Data Availability

No datasets were generated or analysed during the current study.

## Abbreviations

MTT: (3-[4,5-dimethylthiazol-2-yl]-2,5 diphenyl tetrazolium bromide)
AChE: Acetylcholinesterase
DLS: Dynamic Light Scanning
EV: Extracellular vesicles
MVBs: Multivesicular bodies
MMP: Matrix Metalloproteinase
CAFs: cancer-associated fibroblasts
AMPK: AMP-activated protein kinase
BSA: Bovine serum albumin
H3K27me3: Histone H3 at lysine 27
MSLN: Mesothelin
STAT: Signal Transducer and Activator Protein
IL6: Interlukin6
VEGF: Vascular endothelial growth factor
TGFb1: Transforming Growth Factor beta1
FBS: Fetal Bovine serum
IC50: Inhibitory Concentration 50
OD: Absorbance Density
PCR: Polymer Chain Reaction
MVBs: Multivesicular Bodies
